# The Home Observation of Periconceptional Exposures (HOPE) study, a prospective cohort: aims, design, recruitment and compliance

**DOI:** 10.1186/s12940-016-0153-9

**Published:** 2016-06-08

**Authors:** Christina A. Porucznik, Kyley J. Cox, Karen C. Schliep, Diana G. Wilkins, Joseph B. Stanford

**Affiliations:** Office of Cooperative Reproductive Health, Division of Public Health, Department of Family and Preventive Medicine, University of Utah, 375 Chipeta Way, Suite A, Salt Lake City, UT 84108 USA; Center for Human Toxicology, University of Utah, 30 South 2000 East, Salt Lake City, UT 84112 USA; Department of Pathology, University of Utah, 15 North Medical Drive East, Suite 1100, Salt Lake City, 84112 USA

**Keywords:** Fecundity, Preconception, Endocrine disrupting chemicals, Ovulation, Semen, Research participant recruitment

## Abstract

**Background:**

To examine transient environmental exposures and their relationship with human fecundity, exposure assessment should occur optimally at the time of conception in both members of the couple.

We performed an observational, prospective cohort study with biomonitoring in both members of a heterosexual couple trying to conceive. Couples collected urine, saliva, and semen specimens for up to two menstrual cycles on days corresponding to the time windows of fertilization, implantation, and early pregnancy, identified based on the woman’s observations of her cervical fluid.

**Results:**

Three hundred nine eligible couples were screened between 2011 and 2015, of which 183 enrolled. Eleven couples (6.0 %) withdrew or were lost to follow up. The most successful and cost effective recruiting strategies were word of mouth (40 % of participating couples), posters and flyers (37 %), and targeted Facebook advertising (13 %) with an overall investment of $37.35 spent on recruitment per couple. Both men and women collected ≥97.2 % of requested saliva samples, and men collected ≥89.9 % of requested semen samples. Within the periovulatory days (±3 days), there was at least one urine specimen collected by women in 97.1 % of cycles, and at least one by men in 91.7 % of cycles. Daily compliance with periovulatory urine specimens ranged from 66.5 to 92.4 % for women and from 55.7 to 75.0 % for men. Compliance was ≥88 % for questionnaire completion at specified time points.

**Conclusions:**

Couples planning to conceive can be recruited successfully for periconceptional monitoring, and will comply with intensive study protocols involving home collection of biospecimens and questionnaire data.

## Background

A growing body of evidence suggests that environmental exposures during the sensitive windows of the periconceptional period are of concern for the reproductive health of mother and embryo [1–3]. Related research demonstrates that environmental contamination by endocrine disrupting chemicals during the fetal period affects virtually all organ systems in fetal development and throughout subsequent life [4–6]. Transient exposures may have critical influences on fertility, spontaneous abortion, and embryonic development, but limitations in exposure assessment hamper our ability to understand these effects. To monitor transient exposures at or near the time of conception, the time of ovulation must be identified prospectively and targeted exposure assessment must occur during the relevant developmental windows [7–9].

Conception (fertilization in vivo) is a highly-timed, couple-based process [10]. A prospective design that follows couples longitudinally beginning prior to conception and through a pregnancy or pregnancy loss, although methodologically challenging, allows the investigator to capture exposure data during the time-sensitive fertile window and to understand the temporal ordering between exposures and outcomes [8, 11, 12]. Home collection of data and biological specimens by study participants can minimize participant burden while still assessing exposure during the most time-sensitive periods of the fertile window, ovulation, and implantation [13, 14]. Women’s self-observation of cervical fluid patterns has been repeatedly validated as an effective approach for identifying the approach and timing of ovulation [15–17] (i.e., the fertile window [18, 19]), but this approach has not been applied to studies assessing environmental exposures in couples trying to conceive.

We designed and successfully implemented a prospective pregnancy study with home-based collection of data and biological specimens to achieve several aims: (1) conduct prospective, periovulational biomonitoring in a preconception cohort using a novel strategy of self-collection of biological specimens timed according to women’s observation of their own fertility signs among couples with normal fertility; (2) evaluate the association between prospectively measured male and female environmental exposures (primarily bisphenol-A (BPA) and disinfection-by-products) and semen quality and time to pregnancy; and (3) quantify the degree of misclassification bias generated by retrospective exposure assessment methods in common use (participant self-report of past exposure using a recall questionnaire and/or archival environmental monitoring data) compared to concurrent periovulational biomonitoring and report of exposures. This paper addresses our first aim and describes design and implementation of the study protocol. We assess the methodological soundness of our approach by evaluating recruitment, cost of recruitment, eligibility, enrollment, and compliance with collection of appropriately timed biospecimens.

## Methods

### Study design, eligibility, and recruitment

The Home Observation of Periconceptional Exposures (HOPE) study is an observational, prospective cohort study designed to accomplish home-collected, individual-level biomonitoring among heterosexual couples during the sensitive windows of conception, implantation, and very early pregnancy. The University of Utah Institutional Review Board approved the study and written informed consent was obtained from all participants prior to enrollment. Couples were recruited and followed for up to 12 months between 2011 and 2015. Screening was conducted with an online questionnaire (Qualtrics, Provo, Utah, USA) with each member of the couple individually screened to ensure that s/he met eligibility criteria. The inclusion criteria were ages 18–35 for women, ages 18–40 for men, both in a heterosexual relationship, and planning to start trying to conceive within three months of study enrollment. Couples needed to reside within one hour of the study office and be able to respond to study questionnaires and instructions in English. Female exclusion criteria included fewer than nine menstrual flows in the previous twelve months (unless due to breastfeeding or use of an intrauterine device (IUD) with a subsequent return to normal menses); use of implantable hormonal contraception in the previous two months; use of injectable medroxyprogesterone acetate (Depo-Provera®) in the last twelve months. Women were also excluded if they had previously performed daily tracking of cervical fluid but had been unable to identify ovulation due to observations of continuous fluid, no fluid, or unchanging fluid patterns. Couples were excluded if either member of the couple had a previous diagnoses of infertility, subfertility, or a condition that might affect fertility such as diagnosed polycystic ovary syndrome (PCOS) or endometriosis without a subsequent pregnancy among women, and low sperm count among men, or had ever been unsuccessful in conceiving a child after a year or more of regular sexual intercourse without contraception. Our recruitment goal was 300 couples, which we estimated would be a feasible target while providing opportunity to detect variation in time to pregnancy.

Several strategies were implemented to recruit participant couples, including newspaper advertisements, flyers placed around the community and on university and college campuses, targeted Facebook advertising, targeted Google AdWords advertising, campus-wide email listservs, and word of mouth. The online screening questionnaire included a question to determine recruitment method for each inquiring individual. Participants were encouraged to refer friends to the study and were compensated $10 for every referred couple successfully enrolled up to three referrals.

Couples who met the inclusion criteria and consented to participate met with a member of the study staff for an in-person enrollment that took place at the couple’s convenience either in their home, or in a mutually convenient location with sufficient privacy. During enrollment the study staff explained the study procedures, demonstrated use of the online data entry system, explained the daily fertility charting, obtained height and weight measurements, collected hair snips, and explained or demonstrated techniques for home collection of biospecimens. Throughout the study, staff communicated with couples on an individualized basis, via their preferred method (e.g. phone, text, or email).

### Daily fertility chart

**Fig. 1:**
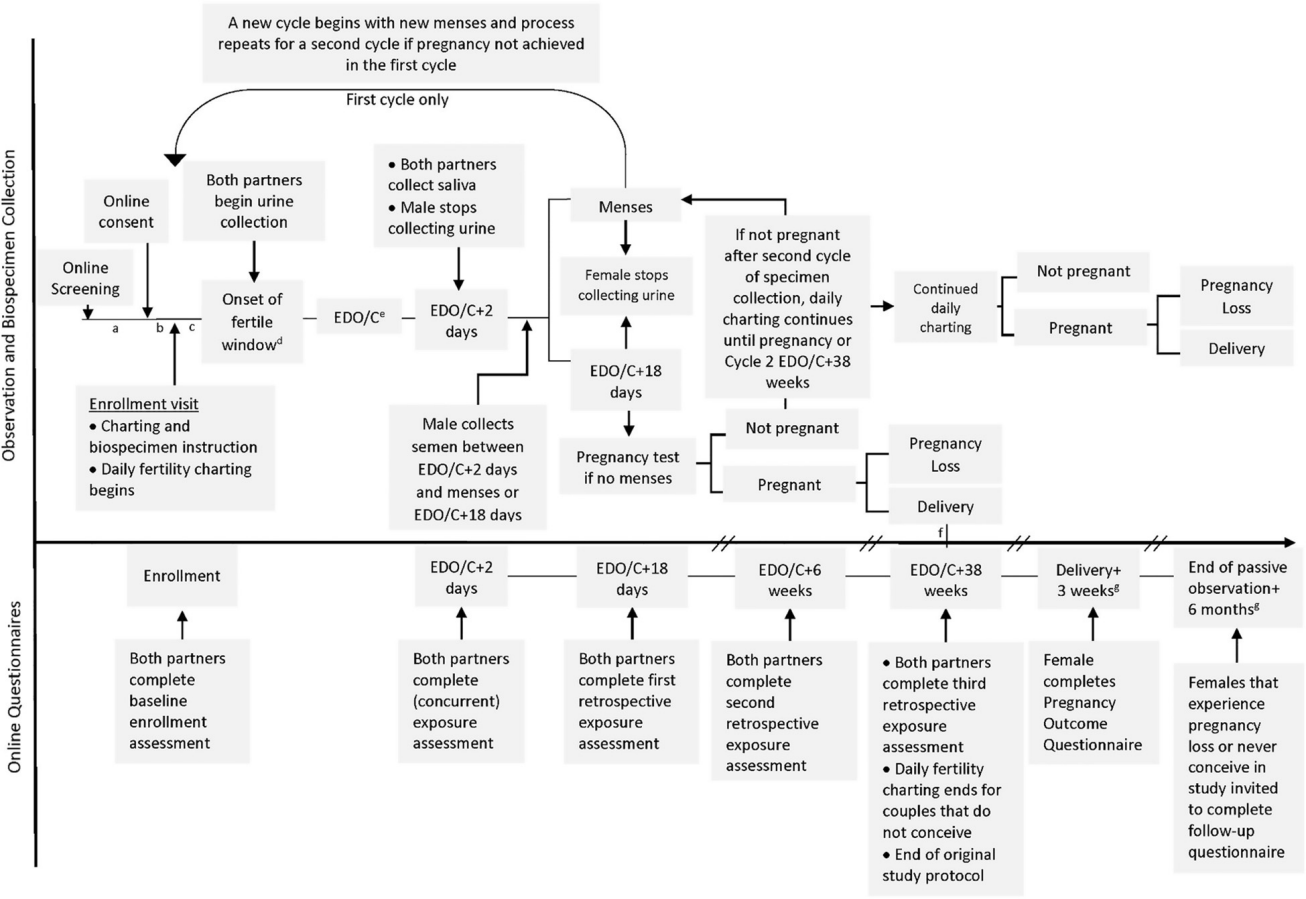
Timeline of study procedures and time-varying data collection. ^a^Mean time between online screening and online consent was 9.5 ± 19.2 days (Range 0–163). ^b^Mean time between online consent and enrollment was 9.9 ± 11.7 days (Range 0–68). ^c^Mean time between enrollment visit and onset of fertile window was 9.8 ± 13.7 days (Range 0–76). ^d^First day of fertile-quality cervical fluid observed by the woman marks the beginning of the fertile window. ^e^Peak Day of fertility according to Peak Day Algorithm (last day of best quality cervical fluid in mid-cycle). ^f^If pregnancy occurred in Cycle 1, delivery estimated to take place at approximately the same time as Cycle 1 EDO/C + 38 weeks. If pregnancy occurred in Cycle 2, delivery estimated to take place at approximately the same time as Cycle 2 EDO/C + 38 weeks. ^g^These two questionnaires were added to the study protocol with IRB approval after recruitment began

The timeline of study procedures for each participating couple was based around the woman’s observations of her fertile window and menstrual cycle, as displayed in Fig. 1. The enrollment visit was conducted during approximately the first ten days of the menstrual cycle, and women were asked to start their daily fertility chart at that time. This timing was designed to rule out pre-existing pregnancy and to observe the estimated day of ovulation or conception (EDO/C) for the first cycle in most instances. This procedure was implemented early in the study after three couples were enrolled when they were found later to be already pregnant. Women were taught daily fertility charting to identify ovulation and the fertile window based on our previously validated approach, the Peak Day Method [20], a simplified method for observing and recording “highly fertile” cervical fluid observed externally as a vaginal discharge, with basal body temperature. The initial appearance of one or more of the “highly fertile” cervical fluid characteristics (clear, stretchy, and/or slippery fluid) was used to identify prospectively the beginning of the fertile window and the approach of the EDO/C. The fertility chart also included daily assessment of lifestyle exposures including intercourse, medications, tobacco, alcohol, caffeine, stress, and illness. Women were asked to record these exposures daily for the entire cycle and men recorded the same exposures daily during the fertile window. Completed fertility charts were either picked up by a member of the study staff or returned by mail using a pre-paid return envelope.

### Active and passive observation periods

The active observation period began in the first cycle after enrollment in which the couples planned to try to conceive and continued for up to two menstrual cycles in which the couple continued to report intention to conceive and an EDO/C was observed. If the couple did not achieve pregnancy within the first two active observation cycles, fertility charting (including the daily exposure questions) continued until the completion of the passive observation period. For couples who conceived in the first cycle of active observation, the end of the passive observation period was Cycle 1 EDO/C + 38 weeks. For all other couples the end of the passive observation period was Cycle 2 EDO/C + 38 weeks regardless of pregnancy status.

### Concurrent and retrospective online questionnaires

In addition to the exposure questions on the daily fertility charts, concurrent (once at EDO/C +2 days) and retrospective (one time each at EDO/C + 18 days, EDO/C + 6 weeks, and EDO/C + 38 weeks) online exposure assessment questionnaires were completed related to the first two complete cycles of active observation, or only the first cycle if the couple conceived during Cycle 1. Questionnaires were completed through a secure response-interface (Qualtrics, Provo, Utah) and were designed to assess exposure to food, water, and lifestyle factors during the week of the EDO/C. The timing of retrospective assessments were selected to correspond to specific time points during pregnancy when early exposure may be assessed: EDO/C + 18 days corresponds to the day when a woman may notice delayed menses; EDO/C + 6 weeks corresponds to when a woman may have a first prenatal appointment (approximately 8 weeks following her last menstrual period); EDO/C + 38 weeks corresponds to when a woman would be expected to deliver a baby. However, questionnaires were administered to everyone in the study, whether or not they became pregnant within the active observation period (first two cycles).

Women with live births resulting from conceptions that occurred during the study were asked to complete an additional questionnaire 3 weeks after the estimated date of delivery to assess pregnancy outcomes, and to provide medical release of information forms to allow study staff to access the prenatal and obstetrical medical records. Women who did not conceive during the study were contacted six months after study completion and asked to complete a questionnaire designed to ascertain any pregnancy or medical treatment that may have occurred beyond the study observation period [21].

### Biospecimen collection

Participants were instructed to perform biospecimen collection during the first two cycles of active observation. Men and women both were asked to collect daily first-morning urine samples (first void upon waking) from the first day of fertile-quality cervical fluid throughout the fertile window until the EDO/C + 2 days. Men were asked to discontinue collecting after EDO/C + 2 days (the end of the biologically relevant time period for conception), and women were asked to continue to collect for the remainder of the menstrual cycle until the onset of the next menses or until she had a positive home pregnancy test (QuickVue, Quidel, San Diego, CA, USA) at EDO/C + 18 days. In cases where first-morning samples were not collected, participants were instructed to collect later in the day and mark the specimen to indicate that it was not first-morning. Urine was collected in four-ounce polypropylene specimen cups then transferred to 50 mL polypropylene tubes that were placed in the participants’ home freezer until the end of the menstrual cycle. At the end of each cycle, a member of the study staff collected the samples from the participants and transported them to the laboratory.

Both members of the couple completed a saliva specimen using a Quantisal Oral Fluid Collection Device (Immunalysis, Pomona, CA, USA) on EDO/C + 2 days. These specimens were retrieved by study staff at the end of each cycle.

A semen sample from the man was collected at home through intercourse after EDO/C + 2 days but before the onset of menses of EDO/C + 18 days using a Male-FactorPak™ semen collection device (Apex Medical Technologies, Inc., San Diego, CA, USA) [22]. The semen sample was frozen in the participant’s home freezer and picked up by staff at the end of the cycle. Abstinence prior to semen sample collection was not required but acts of intercourse were to be tracked on the daily fertility chart.

### Compensation

Couples were compensated up to $400 USD as follows: $15/cycle for completion of two fertility charts and EDO/C + 2 day questionnaires (women); $20/cycle for semen samples and completion of the EDO/C + 2 day questionnaire (men); $10/cycle/person for saliva samples; $20/cycle/person for urine samples; $7.50/cycle/person for EDO/C + 18 day questionnaires; $12.50/cycle/person for EDO/C + 6 week questionnaires; $15/cycle/person for EDO/C + 38 week questionnaires; $20/person study completion bonus; $10/couple for acquaintance referral up to three. Couples who conceived in the first cycle of study participation were compensated what they would have received had they participated in a second cycle to avoid any incentive to delay conception in order to increase compensation [23].

### Statistical analyses

Descriptive statistics were calculated to summarize cohort characteristics, biospecimen collection compliance, and questionnaire completion using SAS software (SAS version 9.4, SAS Institute, Inc., Cary, NC, USA).

## Results

**Fig. 2:**
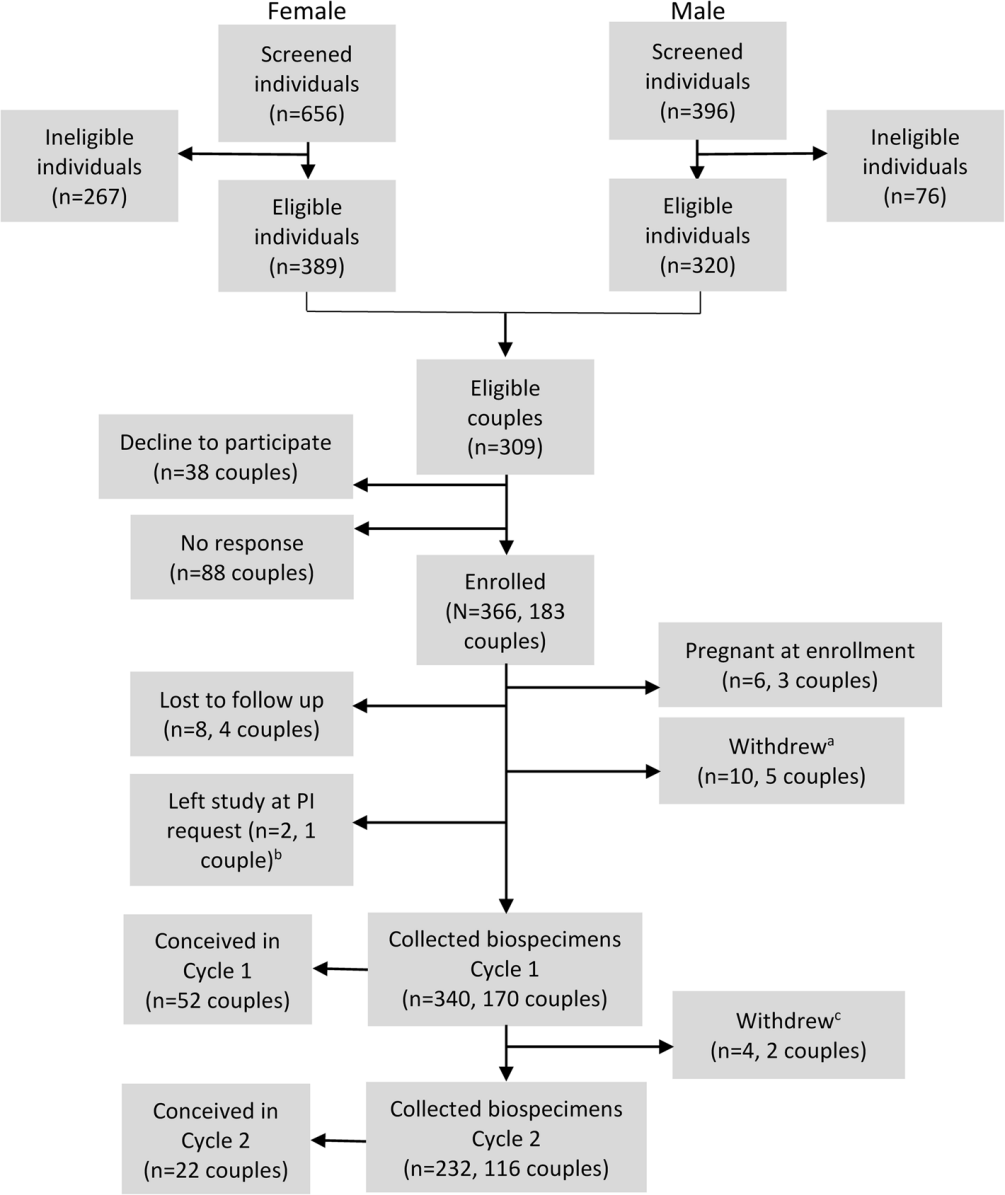
Screening, eligibility, enrollment, and biospecimen completion. ^a^Four of the five couples withdrew due to changed pregnancy intentions and one couple withdrew due to concerns about fertility. ^b^Asked to leave the study due to uninterpretable cervical fluid without recognizable estimated day of ovulation. ^c^One couple withdrew due to changed pregnancy intentions and the other due to stress

One thousand fifty-two individuals (656 women, 396 men) completed the online screening questionnaire with 309 couples meeting the eligibility criteria (Fig. 2). Of the eligible couples, 183 (59.2 %) enrolled, 38 (12.3 %) subsequently declined to participate, and 88 (28.5 %) did not respond to further communication. The most frequent reasons for women being ineligible were being previously being unable to conceive (54, 20.2 %), being already pregnant (38, 14.2 %), using an implantable contraceptive in the past two months (34, 12.7 %), or because of a previous infertility or subfertility diagnoses (32, 12.0 %) (Table 1). When a man was ineligible it was predominantly because his partner was ineligible (56, 73.7 %). In cases where an individual was initially ineligible at inquiry due to a reason that was likely to change over time (i.e., not trying within three months, implantable contraceptive within last two months, fewer than nine menstrual flows due to pregnancy or breastfeeding without subsequent return to normal menses), the individual was asked if he or she would like to be contacted again in the future by study staff to reassess eligibility. Following the repeat contact of 95 individuals, 30 were found to be eligible and enrolled in the study, 16 were still ineligible, 8 declined to participate, and 41 did not respond. Among couples who enrolled, the first member of the couple to complete the screening was most frequently the woman (*n* = 141, 77.0 %). A small proportion of enrolled couples failed to complete the first two cycles of active observation because they either withdrew or were lost to follow-up (*n* = 11 couples, 6.0 %) (Fig. 2).

**Table 1:** Reasons for initial ineligibility by sex^a^

Reason for ineligibility	Male (*n* = 76) n (%)	Female (*n* = 267) n (%)
Age	4 (5.3)	14 (5.2)
>1 h drive from Salt Lake City	1 (1.3)	0
Unable to respond to questionnaires in English	0	0
Not planning to conceive within 3 months^c^	3 (3.9)	32 (12.0)
Medroxy-progesterone acetate injection in last 12 months	NA	14 (5.2)
Implantable contraceptive^bc^	NA	34 (12.7)
Fewer than 9 menstrual flows in the past year^c^	NA	29 (10.9)
Unable to conceive after a year of regular, unprotected intercourse	6 (7.9)	54 (20.2)
Previous infertility or subfertility diagnosis	2 (2.6)	32 (12.0)
Continuous or indistinguishable cervical mucus patterns in previous charting	NA	10 (3.8)
Separated from partner	1 (1.3)	1 (0.4)
Screening incomplete	3 (3.9)	1 (0.4)
Partner ineligible	56 (73.7)	8 (3.0)
Already pregnant	NA	38 (14.2)

*Abbreviations*: NA (not applicable)

^a^Eligibility assessed by online screening questionnaire

^b^Implanon® or intrauterine device

^c^In these cases, individuals may have been contacted later to reassess eligibility

Recruitment costs were assessed by comparing recruitment yield obtained on enrollment questionnaires and budget. Excluding word of mouth that occurred from the spouse or partner, posters and flyers were the most successful recruitment method for initiating screenings (*n* = 246, 23.4 %), but not the most cost-efficient for enrollment ($51.58 per couple enrolled) (Table 2). Referrals from friends or family were also successful in generating screenings (*n* = 207, 19.7 %) and were the most cost-efficient method ($10.00 per couple enrolled). Similarly cost-efficient was targeted Facebook advertising (*n* = 167, 15.9 %) with a cost of $33.31 per enrolled couple. Direct recruitment and advertising cost calculations were calculated for couples rather than individuals by restricting to the index member of the couple (the first member of the couple to complete a screening).

**Table 2:** Efficiency and cost of recruitment by recruitment method

Recruitment Method	Total Screened n(%)	Eligible Screened n(%)	Enrolled n(%)	Enrolled (index^e^ only) n(%)	Cost	Investment per couple enrolled
Word of Mouth						
Spouse/Partner	313 (29.8)	254 (35.8)	149 (40.7)	NA	$0.00	$0.00
Friend/Relative	207 (19.7)	135 (19.0)	88 (24.0)	71 (38.8)	$710.00^d^	$10.00
Healthcare Professional	15 (1.4)	8 (1.1)	3 (0.8)	3 (1.6)	$0.00	$0.00
Web/Email						
Email or newsletter	43 (4.1)	25 (3.5)	8 (2.2)	8 (4.4)	$0.00	$0.00
Facebook	167 (15.9)	95 (13.4)	25 (6.8)	24 (13.1)	$799.44	$33.31
Webpage	28 (2.7)	13 (1.8)	5 (1.4)	5 (2.7)	$0.00	$0.00
Google AdWords	2 (0.2)	0	0	0	$145.00	NA
Local news agency website^a^	4 (0.4)	3 (0.4)	0	0	$1,000.00	NA
Television^b^	7 (0.7)	3 (0.4)	2 (0.5)	2 (1.1)	$0.00	$0.00
Magazine/Newspaper^c^	12 (1.1)	7 (1.0)	3 (0.8)	3 (1.6)	$706.00	$235.33
Posters/Flyers	246 (23.4)	166 (23.4)	83 (22.7)	67 (36.6)	$3,474.18	$51.85
Total	1052	709	366	183	$6,834.62	$37.35

Eight screened respondents (0.8 % of total respondents) did not select a recruitment method and are not included in this table

*Abbreviations*: *NA* not applicable

^a^Deseret News

^b^Principal Investigator interviewed on local news

^c^Utah Family, Catalyst (local magazines)

^d^Cost from compensation given to active participants for referrals

^e^The index person is the initial member of the couple to complete a screening questionnaire

The mean age of female participants was 27.1 ± 3.6 years and of male participants was 28.5 ± 3.8 years (Table 3). The majority of participants (318, 86.9 %) were Caucasian and 23 (6.3 %) were Hispanic. Over half of men (*n* = 109, 59.5 %) and over one-third (*n* = 69, 37.7 %) of women were overweight or obese (BMI ≥ 25.0 kg/m^2^). This population was highly educated with 60.4 % (221) college graduates and 32.5 % (119) with some college education (1–3 years). Over one-third of the cohort (*n* = 131, 35.8 %) had a household income between $40,000 and $74,999 annually. Most participants were never smokers (325, 88.8 %).

**Table 3:** Characteristics of study participants

	All participants (*n* = 366)	Male (*n* = 183)	Female (*n* = 183)
	mean ± SD, or n (%)	mean ± SD, or n (%)	mean ± SD, or n(%)
Age^a^	27.7 ± 4.0	28.5 ± 3.8	27.1 ± 3.6
< 25	69 (18.9)	22 (12.0)	46 (25.1)
25–35	276 (75.4)	145 (79.2)	131 (71.6)
> 35	9 (2.5)	9 (4.9)	0
Missing	12 (3.3)	7 (3.8)	6 (3.3)
BMI^b^	26.1 ± 5.6	27.2 ± 5.8	24.9 ± 5.0
< 18.5	9 (2.5)	2 (1.1)	7 (3.8)
18.5–24.9	178 (48.6)	71 (38.8)	107 (58.5)
25.0–29.9	104 (28.4)	64 (34.9)	40 (21.9)
≥ 30	74 (20.2)	45 (24.6)	29 (15.8)
Missing	1 (0.3)	1 (0.6)	0
Race			
Caucasian	318 (86.9)	156 (85.3)	162 (88.5)
Other/Multiracial^c^	40 (10.9)	23 (12.6)	17 (9.3)
Missing	8 (2.2)	4 (2.2)	4 (2.2)
Hispanic			
Yes	23 (6.3)	12 (6.6)	11 (6.0)
No	334 (91.3)	166 (90.7)	168 (91.8)
Missing	9 (2.5)	5 (2.7)	4 (2.2)
Annual Income^d^			
< $20,000	44 (12.0)	-	-
$20,000–$39,000	109 (29.8)	-	-
$40,000–$74,999	131 (35.8)	-	-
$75,000–$99,000	39 (10.7)	-	-
≥ $100,000	26 (7.1)	-	-
Missing	17 (4.6)	-	-
Employment			
Employed for wages	230 (62.8)	113 (61.8)	117 (63.9)
Self-employed	13 (3.6)	6 (3.3)	7 (3.8)
Homemaker	29 (7.9)	1 (0.6)	28 (15.3)
Student	77 (21.0)	54 (29.5)	23 (12.6)
Unemployed/Other^e^	7 (1.9)	4 (2.2)	3 (1.6)
Missing	10 (2.7)	5 (2.7)	5 (2.7)
Education			
High School/GED	16 (4.4)	10 (5.5)	6 (3.3)
Some college (1–3 years)	119 (32.5)	68 (37.2)	51 (27.9)
College (>4 years, graduate)	221 (60.4)	100 (54.6)	121 (66.1)
Missing	10 (2.7)	5 (2.7)	5 (2.7)
Smoking			
Never	325 (88.8)	156 (85.3)	169 (92.4)
Former	21 (5.7)	16 (8.7)	5 (2.7)
Current	6 (1.6)	5 (2.7)	1 (0.5)
Missing	14 (3.8)	6 (3.3)	8 (4.4)

*Abbreviations*: SD (standard deviation), BMI (body mass index)

^a^Age in years

^b^Body Mass Index: weight (kg)/height (m)2 (measured at enrollment)

^c^Includes Asian, Black/African American, Pacific Islander, American Indian/Alaskan Native

^d^US Dollars, combined household income for both partners

^e^Includes out of work, retired, unable to work

Compliance with collection of saliva and semen samples across both active cycles was as follows: women’s saliva 280/286 = 97.9 %; men’s saliva 278/286 = 97.2 %; semen 257/286 = 89.9 %. Compliance with collection of daily urine samples was also high with specific metrics for each cycle day from three days before to three days after the EDO/C presented in Table 4. Among all active cycles for which we have an identifiable EDO/C, a urine specimen was collected on the EDO/C by the woman in 239 (86.6 %) cycles and by men in 208 (75.4 %) cycles. For active cycles, 97.1 % had at least one urine sample collected by women, and 91.7 % had at least one urine sample collected by men within the perivoluatory days (EDO/C ± 3 days).

**Table 4:** Urine collection completion relative to estimated day of ovulation with or without conception (EDO/C)

	EDO/C-3^a^	EDO/C-2^a^	EDO/C-1^a^	EDO/C	EDO/C + 1	EDO/C + 2	EDO/C + 3
All Cycles							
Female	124/185 (67.0)	175/234 (74.8)	216/262 (82.4)	239/276 (86.6)	249/276 (90.2)	258/276 (93.5)	257/276 (93.1)
Male	103/185 (55.7)	146/234 (62.4)	191/262 (72.9)	208/276 (75.4)	207/276 (75.0)	164/276 (59.4)	72/276 (26.1)^b^
Conception Cycles							
Female	37/53 (69.8)	51/65 (78.5)	62/73 (84.9)	64/74 (86.5)	66/74 (89.2)	70/74 (94.6)	65/74 (87.8)
Male	32/53 (60.4)	42/65 (64.6)	52/73 (71.2)	58/74 (78.4)	55/74 (74.3)	48/74 (64.9)	23/74 (31.1)^b^

^a^Denominator varies due to timing of enrollment and timing of the start of the fertile window. In some cases the first day of highly fertile cervical fluid occurred fewer than three days prior to the EDO/C and in some cases enrollment occurred fewer than three days prior to the EDO/C

^b^Males were instructed to discontinue collecting at Peak + 2 so specimens collected on Peak + 3 were extra

Completion rates for concurrent and retrospective questionnaires are reported in Table 5. Compliance was ≥88 % for all concurrent and retrospective online questionnaires. When stratified by sex, completion rates for females ranged from 90 to 98 % and for males ranged from 85 to 94 %. Fertility charts were collected for 623 (80.6 %) cycles.

**Table 5:** Concurrent and retrospective questionnaire completion by cycle and sex

	EDO/C + 2 days n(%)	EDO/C + 18 days n(%)	EDO/C + 6 weeks n(%)	EDO/C + 38 weeks n(%)
Cycle 1				
Combined (*n*=340)	324 (95)	325 (96)	315 (93)	308 (91)
Female (*n*=170)	164 (96)	166 (98)	164 (96)	157 (92)
Male (*n*=170)	160 (94)	159 (94)	151 (89)	151 (89)
Cycle 2				
Combined (*n*=232)	208 (90)	207 (89)	203 (88)	208 (90)
Female (*n*=116)	108 (93)	107 (92)	104 (90)	106 (91)
Male (*n*=116)	100 (86)	100 (86)	99 (85)	102 (88)

*Abbreviations*: EDO/C estimated day of ovulation or conception

## Discussion

Our findings support a growing body of evidence that both women and men planning pregnancy can be recruited and will comply with protocols requiring the collection of biospecimens and questionnaire data in the periconception window [12, 13, 23–27]. Similar to prior studies, the most common reasons for ineligibility were having already become pregnant or subfertility, highlighting the importance of rapid recruitment among couples intending to conceive [23, 27, 28]. We successfully enrolled 59.2 % of couples who screened eligible, a proportion similar to other studies [13, 24, 27]. Participants were self-selected volunteers and thus, no direct denominator is available for the target population at risk of or planning pregnancy. Word of mouth referrals from current participants (with modest recruitment incentives) proved to be the most efficient recruitment tool. Targeted Facebook ads, an innovative recruitment strategy, were productive and highly cost-effective in our study, similar to another recent preconception enrollment study [27]. Facebook allowed us to target our advertisement to appear to select individuals based on marital status, age, sex, and location. Posters and flyers generated nearly one-quarter of our total screenings but were less cost-effective. In terms of strategic planning for recruitment, targeting women had a greater yield in generating screenings.

Participants in our study were predominantly white and well educated. There was a low proportion of current smokers (<2 %), and the mean age for women was 27.1 years. While the lack of racial and ethnic diversity may be a limitation, the absence of smoking allows for less confounding in examining the impact of other environmental and lifestyle exposures on reproductive outcomes.

Retention of participants despite the intensive study protocol was high with a dropout rate of only 6.0 % (11 couples). This rate is lower compared to reported rates from other prospective cohorts of couples trying to conceive in Denmark (8 %) [24], France (36 %) [29], Michigan and Texas (26 % and 27.2 %, respectively) [13], at four centers in the United States (12.2 %) [28], through internet-based methods (19 %) [27], and at seven European centers (25 %) [30], but higher than a study in Washington, DC (4 %) [25]. We believe this high retention rate was related to effective relationships that study staff built with participant couples, flexibility in communication, the convenience of home based visits, and study compensation commensurate with the level of intensity of data collection.

Compliance with collection of semen samples was high over two cycles (89.9 %). Comparatively, 64 % of monthly semen samples for up to six cycles were received in Denmark [24], and the LIFE Study [31] reports 94 % compliance with semen collection at baseline and 77 % compliance with semen collection for a second semen sample; both studies collected via masturbation. Investigators may wish to consider the utility of home-based collection of semen samples through intercourse with semen collection devices to increase response rate, depending on the cultural context; although study objectives and analytical limitations of frozen specimens versus fresh specimens must be considered.

Comparison of compliance with collection of urine to other preconception studies is difficult as study designs vary widely. Bonde et al. [24] report 84 % compliance with women submitting daily urine on days one through ten of each menstrual cycle up to six cycles or until pregnancy, and men with 59 % compliance in submitting one to two pre- or post-shift urines monthly during those cycles. Zinaman et al. [25] requested women to submit daily morning urine samples for three cycles; compliance was >90 %. Buck-Louis et al. [13] requested three spot samples per woman with compliance ranging from 77 to 100 %, and two spot urine samples per man with compliance ranging from 94 to 100 %. Wilcox et al. [26] reported 98 % compliance among 221 women collecting daily samples for up to six months or until pregnancy. In this study, compliance with this collection protocol on periovulatory days (EDO/C ± 3 days) ranged from 67 to 94 % for women and from 56 to 75 % for men. Because men must rely on communication from the woman to start urine collection, it is unclear whether compliance among men is lower than women due to a lack of communication between partners or if men were aware but less motivated. Overall compliance with completion of online concurrent and retrospective questionnaires was high, and females consistently had higher completion rates compared to males.

Possible approaches to obtaining urine and other biospecimens in the fertile window include collecting urine every day, which may be more resource intensive, using fixed calendar days, which is much less accurate due to natural variability in the fertile window [32], or using indicators of the fertile window to target sample collection accordingly. Targeting biospecimen collection to the fertile window has been done effectively in prior studies using a handheld, home fertility monitor based on urinary hormones of estrone glucuronide and luteinizing hormone [13, 28, 33]. However, use of a fertility monitor may be expensive for larger studies. In this study, women identified the fertile window based on self-observation of fertile-quality cervical fluid, supplemented and confirmed by basal body temperature measurements [20]. This approach is inexpensive, efficient, and resulted in high compliance in our study.

## Conclusions

Our findings support a growing body of evidence supporting the feasibility of successful completion of preconception cohort studies for assessing environmental exposures during the earliest critical windows of human development around conception and implantation, with excellent retention and high compliance of detailed exposure monitoring (biospecimens and questionnaires). Our results also confirm the emerging importance of targeted online recruitment strategies. Our study is one of a few that have successfully included all of the male partners, a critical component for studying environmental impacts on fecundity and early human development. Finally, we have demonstrated for the first time the effective implementation of a streamlined, cost-efficient approach of women’s self-observation of fertility symptoms (primarily cervical fluid) applied to periconceptional exposure monitoring.

## Abbreviations

BMI, Body mass index; BPA, Bisphenol A; EDO/C, Estimated day of ovulation or conception; HOPE, Home observation of periconceptional exposures; IUD, Intrauterine device; LIFE, Longitudinal investigation of fertility and the environment; PCOS, Polycystic ovarian syndrome; USD, United States dollar
